# An Overview of Distributed Microgrid State Estimation and Control for Smart Grids

**DOI:** 10.3390/s150204302

**Published:** 2015-02-12

**Authors:** Md Masud Rana, Li Li

**Affiliations:** Faculty of Engineering and Information Technology, University of Technology, Sydney Broadway, NSW 2007, Australia; E-Mail: Li.Li@uts.edu.au

**Keywords:** discrete-time algebraic Riccati equations, distributed energy resource, Kalman filter, smart grid, state estimation, wireless sensor networks

## Abstract

Given the significant concerns regarding carbon emission from the fossil fuels, global warming and energy crisis, the renewable distributed energy resources (DERs) are going to be integrated in the smart grid. This grid can spread the intelligence of the energy distribution and control system from the central unit to the long-distance remote areas, thus enabling accurate state estimation (SE) and wide-area real-time monitoring of these intermittent energy sources. In contrast to the traditional methods of SE, this paper proposes a novel accuracy dependent Kalman filter (KF) based microgrid SE for the smart grid that uses typical communication systems. Then this article proposes a discrete-time linear quadratic regulation to control the state deviations of the microgrid incorporating multiple DERs. Therefore, integrating these two approaches with application to the smart grid forms a novel contributions in green energy and control research communities. Finally, the simulation results show that the proposed KF based microgrid SE and control algorithm provides an accurate SE and control compared with the existing method.

## 1. Introduction

Green energy technologies with distributed energy resources (DERs), such as solar cells, photovoltaics and wind power, have recently attracted significant attention in smart grids due to their harmonious relationships with nature, their green impact on the global environment by generating electricity without carbon emissions and their sustainability [1,2]. Combining these DERs of every place, the community can generate sufficient electricity to keep the police department, the phone system, the traffic lights and the community health centre up and running [3,4]. Unfortunately, their power generation patterns are mostly intermittent in nature and distributed over the grid, which creates challenging problems in the control and reliability of the smart grid. Thus, this grid has a strong requisite of an efficient communication infrastructure to monitor and control the DER states (current, voltage and power). This grid can also spread the intelligence of the energy distribution and control system from the central unit to long-distance remote areas, thus enabling accurate state estimation (SE) and wide-area real-time monitoring of these intermittent energy sources [5]. As a result, reliability tools and power system operations totally depend on the results obtained by the SE [6].

### 1.1. An Overview State-of-the-Art Work

In order to estimate the system states, various algorithms and tools have been adopted for smart grids. For instance, static state estimators are extensively applied in smart grids due to the fact that the traditional monitoring technologies, such as supervisory control and data acquisition (SCADA) systems, rely on a single set of measurements [7]. In order to estimate the state of the power system regularly, this process is repeated at suitable intervals of time [8–10]. In addition, the first step toward a dynamic state estimator is taken by Debs and Larson in [11], where a simple state transition model is considered, and it is assumed that the power system is a quasi-static system and that the states change slowly with time. It provides a better performance; however, it requires an accurate dynamic model and incurs relatively more computational complexity. However, in multi-area power systems, several different estimation stages are used [7]. In the local SE stages, the estimators gather measurements from the DERs and estimate the states, which are shared with the neighbouring local estimators. Next, the global estimator combines the local estimation information and re-calculates the states [12,13].

Power system SE frequently uses the weighted least squares (WLS) method that minimizes the sum square of the weighted residuals [14]. The main problem of the WLS method is that the gain matrix may be ill-conditioned. Thereby, the solution may fail to converge, and system states cannot be obtained accurately [15,16]. The numerically ill-conditioned problem is successfully solved by the trust region method with quadratic regulation (QR) factorization, but the convergence problem still exists [17]. Besides, the estimation process of telemetering measurements is treated individually as an additional constraint to the WLS method [18]. Then, the constrained minimization problem is solved by the Lagrange multiplier method [19]. In [18], a similar constrained WLS problem is formulated, where the explicit optimization variables are the measurement residuals. The work in [20] provides a taxonomy of multi-area state-estimation methods, which currently is raising enormous interest, due to their capability of properly tracking the multi-area case and accommodating highly redundant information. In [21], the authors propose a decentralized SE method, where the local estimator estimates the system states of each subregion considering that the border measurements belong to the specific local region. After that, the central coordinator coordinates all local estimators. Afterwards, this decentralization estimation algorithm is improved for the multi-region case without needing any common border buses [22]. Similarly, the central coordinator exchanges information among the neighbouring areas and exploits the structure of the problem to achieve a fast and accurate convergence [23].

A joint state and parameter estimation method in power systems is proposed in [24,25], but it fails to identify any dynamic pattern properly The next breakthrough in dynamic SE comes from [26], which provides an appropriate state transition model. This model uses a Kalman filter (KF) and an exponential smoothing algorithm for state forecast [26,27]. The KF-based SE is widely used in the literature and provides a recursive update of the state during system operation [28]. Furthermore, a robust forecast-aided SE algorithm is presented in [29], which is considered an alternative form of the KF approach. In fact, most of the power system problems in the real word are non-linear. In the literature, the most widely-used nonlinear SE is the extended KF (EKF). However, the unscented KF (UKF) SE is much easier to implement, because there is no need for the linearization and the derivation of Jacobian matrices, so the simulation time is less than that of the EKF SE. In other words, errors are introduced due to the linearization and calculating of the Jacobian matrix, which is a very difficult and error-prone process. Therefore, this UKF SE method converges to the right solution more rapidly, and the results become stable and unbiased.

Recently, a belief propagation (BP)-based static state estimator for the IEEE 4-bus distributed system was proposed in [30]. However, the system states continuously change over time. Interestingly, given the significant concerns regarding global warming and the energy crisis, distributed solar and wind power are going to be integrated into the grid to save energy resources, so that state monitoring is needed in the distributed microgrid. In fact, a BP algorithm for unregulated dynamic SE for a single microgrid is proposed in [31]. Unfortunately, the computational complexity of the BP method is very high, even though the performance is almost the same at a high signal-to-noise ratio. Furthermore, the smart grid is to integrate the multiple DERs into the main grid, which needs to be controlled properly, as it is distributed in remote areas. Another critical challenge is that the performance of the estimation framework for the DER SE has to handle a hybrid system: a continuous dynamic system state with observation noise and discrete information bits in wireless communication systems. Obviously, the introduction of time-varying parameters in the system model is needed for accurate representation of the DER behaviour, leading to more challenging problems in the estimation and control. Therefore, an alternative approach is required to monitor such a system considering the multiple DERs with a flexible sensing network. The key contributions of the paper are summarised in the next subsection.

### 1.2. Main Technical Contributions

Motivated by the above factors and technical challenges, the main objective of this paper is to show how the measurements can be effectively aggregated in the central estimator and to develop a technique to estimate and control the state from the observations in the microgrid incorporating multiple DERs. In this innovative study on microgrid SE and control, this paper proposes to use accuracy-dependent KF for DERs SE and discrete-time linear quadratic regulation (DLQR) to control the DER state with typical communication systems. The main technical contributions of the paper can be summarised as follows:

- First of all, a real microgrid incorporating multiple DERs is modelled as a continuous linear system. Then, it is rewritten as a discrete linear system considering the uncertainty. Flexible and cost-effective smart sensors are deployed to get DER state information, which is transmitted to the nearby base station. In other words, the uniform quantizer at this point maps each observation signal to a sequence of bits, where the information is transmitted through a wireless channel. Based on this infrastructure, a state observer and DLQR control are designed and implemented for the smart grid.
- In order to properly monitor these intermittent energy sources from any place, this paper proposes a novel accuracy-dependent consensus scheme in the standard KF SE method. The proposed approach is able to properly estimate the DER states through minimizing the estimation error based on the current estimation, learning rate parameter and trust covariance matrix. Then, the estimated system states are fed back to the DERs for controlling the state. As the point common coupling (PCC) voltage deviations are increased dramatically, it is necessary to apply a proper control method, so that the PCC voltage deviations are driven to zero; otherwise, it is very dangerous in terms of network stability and the operation of the DERs.
- Finally, this paper proposes a DLQR method to control the state of the microgrid incorporating multiple DERs. The proposed controller can stabilise the voltage deviations of the DERs within 7 seconds, even if there are large disturbances. Therefore, integrating these two approaches with application to the smart grid forms a novel contribution in the green energy and control research communities. The simulation results show that the proposed KF-based microgrid SE and control algorithm provides accurate SE and control compared with the existing method. Based on the proposed estimation and control in the smart grid with a communication infrastructure, customers are willing to use environmentally-friendly DERs to mitigate the impending energy crisis of finite resources. In other words, integration of DERs into the grid can result in several benefits, such as line loss reduction, reduced environmental impacts, peak savings and overall improvement of energy efficiency, reliability and power quality [32]. In fact, customers can also benefit from the DERs to meet their basic daily demand and have better quality of supply at a lower cost. For instance, some developing countries suffer from electricity outages, so they can use these DERs as an essential requirement to meet basic demands; also, 35 percentage of total electricity is generated from DERs in Germany. Combining the DERs of every place, the community can generate sufficient electricity to keep the police department, the phone systems and the public health centre up and running. This work will involve the engineering communities of communication, network and power and will shed light on green smart control and monitoring centre design in future smart grid implementations.

The rest of this paper is organized as follows. A brief overview of the smart grid and a DER model with communication systems are presented in Sections 2 and 3, respectively. In addition, the proposed KF-based dynamic SE scheme and controller design are described in Section 4, followed by the simulation results and discussions in Section 5. Finally, the paper is wrapped-up with conclusions and future work in Section 6.

The following notations are used in this paper: bold face lower and upper case letters are used to represent vectors and matrices, respectively. Furthermore, superscripts x^*^ and **x***^T^* denote the conjugate and transpose of **x**, respectively. Lastly, **I** is the identity matrix.

## 2. An Overview of the Smart Grid Communications

**Figure 1.**
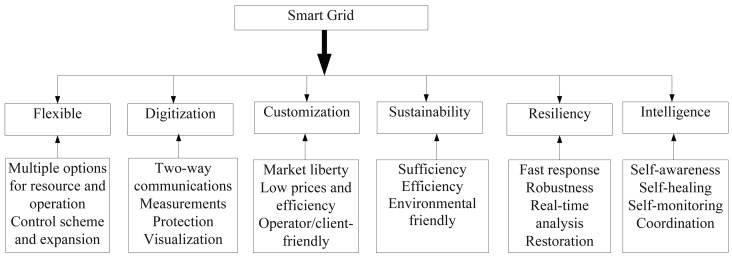
The main characteristics of a smart grid.

The smart grid is a two-way flow of electricity and information between energy producers and consumers, which makes a widely-distributed and automatedly controlled energy delivery network [33]. By exploiting two-way communications, it becomes possible to replace the current power system with more intelligent infrastructures [34,35]. Therefore, the smart grid is seen as a modernization of both transmission and distribution power grids with the features of self-awareness, self-organisation and self-recovery [5,35]. The main characteristics of a smart grid are illustrated in Figure 1.

**Figure 2.**
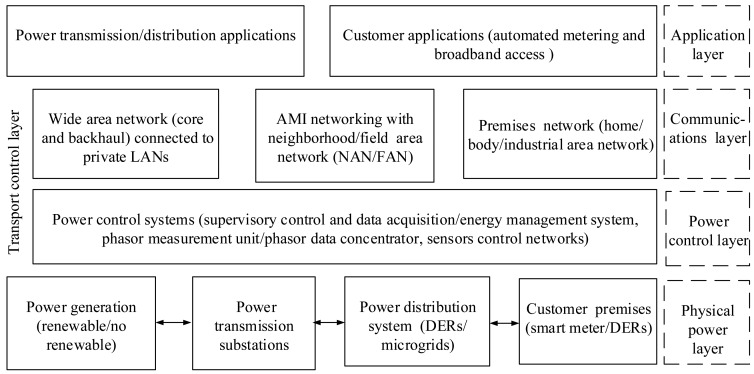
System architecture for the smart grid paradigm.

The smart grid is based on the development of communication infrastructures incorporated into the electrical grid to enhance the information exchange and achieve fully-automated management in power systems. From this point of view, the smart grid architecture is divided into four dominant layers: physical power layer, power control layer, communications layer and application layer [36]. This system architecture for the smart grid paradigm is demonstrated in Figure 2. The physical power layer includes the power generation unit, transmission systems (delivering power from the plants to the substations) and distribution systems (delivering power from the substations to the consumers). The power control layer involves advanced sensing technologies, measurement devices, controls and monitoring equipment, such as smart sensors, phasor measurement units, SCADA and actuators. The communications layer provides reliable, secure and effective information exchange between the layers. Lastly, the application layer supports all of the services provided to the end customers and utilities, such as automated metering and broadband access [34,36].

**Figure 3.**
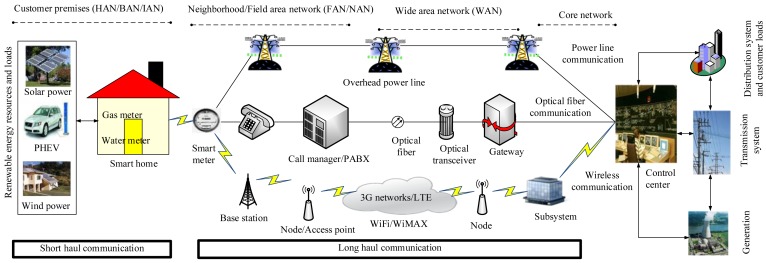
Different communication standards and protocols of a smart grid.

Furthermore, the communications layer has premises networks, neighborhood/field area network (NAN/FAN) and wide area network (WAN). The premises network, such as the home area network (HAN), building area network (BAN) and industrial area network (IAN), provides access to applications in the customer premises. The NAN/FAN provides a relatively long-distance communication link between smart meters, field devices, DERs, customer premises’ networks and WAN. The WAN provides very long-distance communication links between the grid and the utility via a core network and FAN/NAN. However, the coverage area and data rate requirements for the customer premises’ network, FAN/NAN and WAN vary for different communication standards and protocols (wired and wireless). To illustrate, Figure 3 shows different communication standards and protocols of a smart grid. The wireless communications are easier to deploy (especially in remote areas), more flexible and portable than wired networks, such as power line communication (PLC) and optical fiber communication [36]. However, the security, reliability and power consumption are the main problems for this network [37].

Due to the rapid development of wireless communication technologies, these tools could meet the visions of the smart grid and will be widely used in advanced metering infrastructure, demand response, distribution automation, wide area measurement systems and stability control. These technologies, such as third generation (3G), fourth generation (4G) and fifth generation (5G), are bidirectional communication systems with wide coverage and are suitable for widespread terminal access and remote control in the smart grid. A crucial new technology that can assist utilities with their smart grid is 4G long-term evolution (LTE). LTE uses orthogonal frequency division multiple access, making it better able to leverage the wireless spectrum in smart grid applications. Thus, LTE is able to deliver more data, something obviously useful for a utility smart grid. According to the International Telecommunications Union, a 4G network requires a mobile device to be able to exchange data at 100 Mbit/s (download) and 50 Mbit/s (upload); while the LTE-advanced update is expected to offer peak rates up to 1-Gbit/s fixed speeds and 100 Mb/s to mobile users [38]. Moreover, LTE is based on IP architecture, which means fewer network elements and the ability to easily include a larger number of smart grid devices. Being an all-IP architecture, LTE uses a sophisticated quality of service (QoS) control that utilities can leverage to guarantee that incident and emergency data take priority over less critical wireless data. Therefore, LTE is emerging as a potential new revenue stream for utilities. In fact, LTE deployments in conjunction with smart grids in electric utilities are now positioned to offer better customer services, such as high-speed DER data, voice and video, either directly or based on a wholesale model [39], which is clearly changing the broadband landscape.

Interestingly in smart grids, the communication network plays a vital role to support control operations, deliver system state information to the destinations and interact between substations and control centres [40]. In order to realise the smart grid features, one of the prerequisites is the observability of system state information, such as power flows, voltage, currents, phase and frequency across the grid. Therefore, reliable SE is a key technique to fulfil this requirement and, hence, is an enabler for the automation of power grids. The DER model and smart grid communications are presented in the next section.

## 3. DER Model and Smart Grid with Communication Systems

The main goal of this paper is to propose an accuracy-dependent KF-based microgrid SE and control for the smart grid with communication systems. First of all, in order to sense the DER states using sensors, we try to answer the following question: what is the suitable communication infrastructure for sensing, estimating and controlling the microgrid incorporating multiple DERs? This section attempts to answer this question by presenting a real microgrid model incorporating multiple DERs together with a sensing model, uniform quantization and communication systems.

### 3.1. A Microgrid Model Incorporating DERs

**Figure 4.**
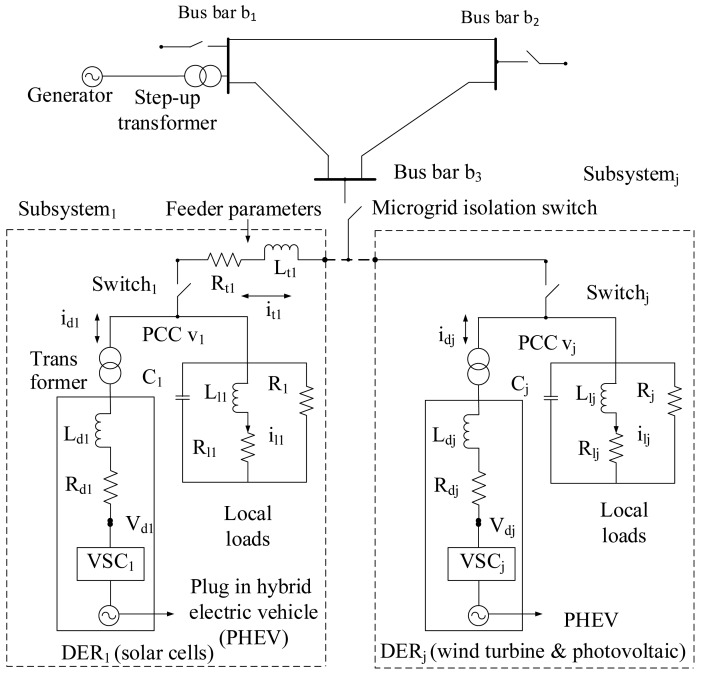
Block diagram of the microgrid incorporating distributed energy resources.

Figure 4 shows the schematic diagram of the multiple DERs connected to the IEEE 3-bus distribution system. The DER is interfaced with the local load through a converter. Additionally, each DER is represented by a DC voltage source in series with a voltage source converter (VSC) and an RLfilter. The inductor opposes changes in the current through it. The resistance can protect the electric circuit. In addition, it contains a step-up transformer between each DER and the load. The local load of each DER is modelled by a three-phase parallel balanced RLCload. Generally, the distributed microgrid can be operated in the grid-connected or the islanded mode based on the status of local circuit breaker of each DER. For the stand-alone operation, the DERs must maintain the voltage level of the local loads within their acceptable values [41]. In this paper, the distributed microgrids are connected to the main grid. To maintain the load voltage, an appropriate control strategy for the autonomous operation of the DERs must be developed. Based on Kirchhoff's voltage law and Kirchhoff's current law, the equations of the DERs can be expressed as:

(1) $$\overset{\cdot }{i_{lj}}=(\upsilon_{j}-R_{lj}i_{lj})/L_{lj}$$

(2) $$\overset{\cdot }{i_{dj}}=(\upsilon_{dj}-R_{dj}i_{dj}-\upsilon_{j})/L_{dj}$$

(3) $$\overset{\cdot }{i_{tj}}=(\upsilon_{j}-R_{tj}i_{tj}-\upsilon_{j+1})/L_{tj}$$

(4) $$\overset{\cdot }{\upsilon_{j}}=(-\upsilon_{j}/R_{j}-i_{lj}+i_{dj}-i_{tj})/C_{j}$$

where *i_dj_*, *i_tj_* and *i_lj_* are the current of *DER_j_*, distribution line *j* and load *j*, respectively. *R_dj_*, *R_tj_* and *R_lj_* are the resistances of the *VSC_j_* filter, distribution line *j* and load *j*, respectively. *L_dj_*, *L_tj_* and *L_lj_* are the resistances of the *VSC_j_* filter, distribution line *j* and load *j*, respectively. *C_j_* is the capacitance, and *υ_j_* is the PCC local bus voltage that needs to be controlled. Forsimplicity, we consider three distributed subsystems (*j* = 1,2 *and* 3). In addition to the above equations, for the last subsystem (e.g., *j* = 3), there is no *υ_j_*_+1_ (e.g., *υ*_4_ = 0) and no transmission line *RL* parameters (*R_t_*_3_ = 0, *L_t3_* = 0 and *i_t_*_3_ = 0).

The dynamic system described by Equations (1)–(4) can be written in the following form:

(5) $$\dot{\text{x}}(t)=\text{Ax}(t)+\text{Bu}(t)+\text{n}(t)$$

where **x** = (*i_l_*_1_ *i_d_*_1_ *i_t_*_l_ *υ*_1_ *i_l_*_2_ *i_d_*_2_ *i_t_*_2_ *υ*_2_ *i_l_*_3_*i_d_*_3_ *υ*_3_)*^T^*, **u** = (*υ_d_*_1_ *υ_d_*_2_ *υ_d_*_3_)*^T^* and

$$\begin{array}{c} \text{A}=\left[\begin{array}{lllllllllll} -R_{l1}L_{l1}^{-1} & 0 & 0 & L_{l1}^{-1} & 0 & 0 & 0 & 0 & 0 & 0 & 0\\ 0 & -R_{d1}L_{d1}^{-1} & 0 & -L_{d1}^{-1} & 0 & 0 & 0 & 0 & 0 & 0 & 0\\ 0 & 0 & -R_{t1}L_{t1}^{-1} & L_{t1}^{-1} & 0 & 0 & 0 & -L_{t1}^{-1} & 0 & 0 & 0\\ -C_{1}^{-1} & -C_{1}^{-1} & -C_{1}^{-1} & -R_{1}^{-1}C_{1}^{-1} & 0 & 0 & 0 & 0 & 0 & 0 & 0\\ 0 & 0 & 0 & 0 & -R_{l2}L_{l2}^{-1} & 0 & 0 & -L_{l2}^{-1} & 0 & 0 & 0\\ 0 & 0 & 0 & 0 & 0 & -R_{d2}L_{d2}^{-1} & 0 & -L_{d2}^{-1} & 0 & 0 & 0\\ 0 & 0 & 0 & 0 & 0 & 0 & -R_{t2}L_{t2}^{-1} & L_{t2}^{-1} & 0 & 0 & -L_{t2}^{-1}\\ 0 & 0 & 0 & 0 & -C_{2}^{-1} & C_{2}^{-1} & -C_{2}^{-1} & -R_{2}^{-1}C_{2}^{-1} & 0 & 0 & 0\\ 0 & 0 & 0 & 0 & 0 & 0 & 0 & 0 & -R_{l3}L_{l3}^{-1} & 0 & L_{l3}^{-1}\\ 0 & 0 & 0 & 0 & 0 & 0 & 0 & 0 & 0 & -R_{d3}L_{d3}^{-1} & -L_{d3}^{-1}\\ 0 & 0 & 0 & 0 & 0 & 0 & 0 & 0 & -C_{3}^{-1} & C_{3}^{-1} & -C_{3}^{-1}R_{3}^{-1} \end{array}\right]\\ \text{B}=\left[\begin{array}{lll} 0 & 0 & 0\\ L_{d1}^{-1} & 0 & 0\\ 0 & 0 & 0\\ 0 & 0 & 0\\ 0 & 0 & 0\\ 0 & L_{d2}^{-1} & 0\\ 0 & 0 & 0\\ 0 & 0 & 0\\ 0 & 0 & 0\\ 0 & 0 & L_{d3}^{-1}\\ 0 & 0 & 0 \end{array}\right], \end{array}$$

Finally, **n**(*t*) is the process distortion due to discretisation and the surrounding ambient conditions of the DERs whose mean is zero and variance is Σ*_n_*. The discretisation of the DER state space model is described in the next subsection.

### 3.2. Discretisation of the DER State Space Model

By applying the Euler formula, Equation (5) can be written in the following discrete form,

(6) $$\text{x}(k+1)={\text{A}}_{d}\text{x}(k)+{\text{B}}_{d}\text{u}(k)+{\text{n}}_{d}(k)$$

where **A***_d_* = *exp*(**A**Δ*t*) ≈ **I** + **A**Δ*t*, ${\text{B}}_{d}=\int_{0}^{\Delta t}\text{exp}(\text{A}\xi )\text{B}d\xi \approx \text{B}\Delta t,$ **n***_d_*(*k*) = Δ*t***n**(*k*) with the variance Σ*_nd_*, Δ*t* is the step size parameter, *exp*(.) is the exponential function, **I** is the identity matrix and **A***_d_* and **B***_d_* are the discrete entities form the continuous version of **A** and **B**, respectively [41]. Therefore, the DER model is discretised, and kis used as the discrete time step, while tis the continuous time. The network architecture for sensing the DER states is described in the next subsection.

### 3.3. Network Architecture for Sensing the DER States

**Figure 5.**
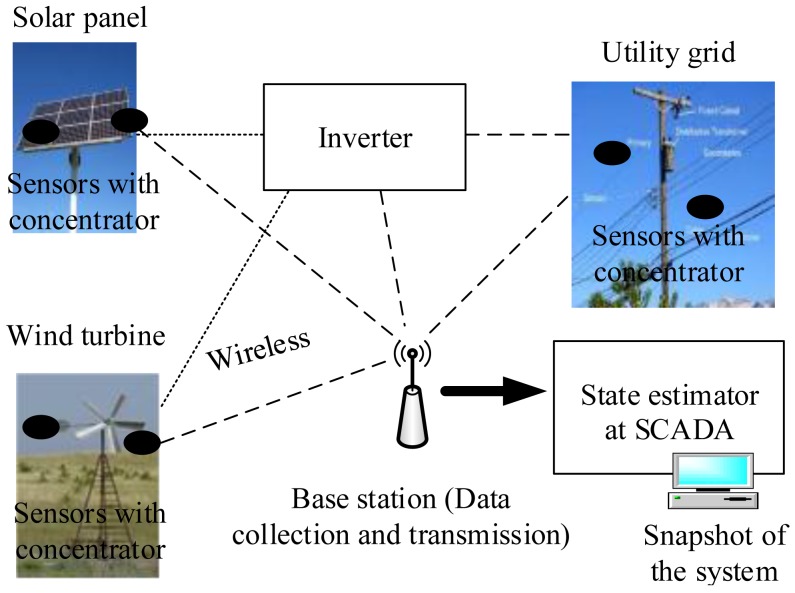
DER state information is coordinated by wireless sensor in the smart grid.

**Figure 6.**
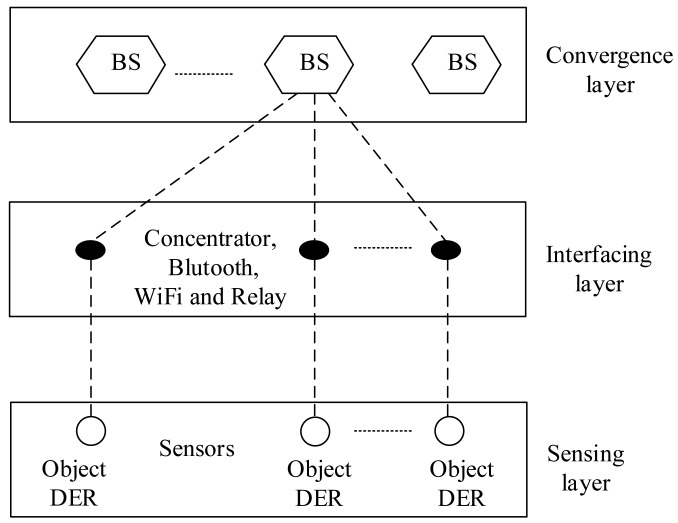
Example of the system framework for smart grids deployment.

Generally, the touted strength of a smart grid is the ability for making its various entities interact via full-duplex communication networks [33]. From this point of view, wireless sensors provide a feasible and cost-effective sensing and communication solution for smart grid applications. Therefore, this enables large-scale deployment of the sensors. To this end, the sensing nodes must efficiently send data over long distances from the DER source to the base station [42,43]. To illustrate, Figure 5 shows a typical sensor setup for DER state monitoring of a smart grid. Usually, sensor devices are mounted on boards attached to the monitored DERs. Moreover, the sensor nodes are organized as a structured one with a concentrator acting as the data collector. Explicitly, each sensor node generally has a radio transceiver, a small microcontroller and an energy source [42,43]. In this way, the sensor nodes communicate with the base station through a wireless data exchange standard, such as concentrator, Bluetooth, wireless fidelity (WiFi) and relay [43]. Next, this collected information at the base station is transmitted to the smart observer at the SCADA system, possibly through a satellite, wireless channel and the Internet. To clarify this, Figure 6 depicts a paradigmatic example of the system framework for a smart grid deployment that includes three layers, *i.e.*, sensing layer, interfacing layer and convergence layer, from bottom to top.

The sensing layer is used to obtain the DER state information. The interfacing layer is formed by a collection of the concentrator, Bluetooth, WiFi and relay node [43]. It is notable that this work uses concentrators to aggregate and forward the DER data. In addition, a concentrator is also installed for each transformer to ensure the communication quality considering transmission loss through the transformer. Finally, the convergence layer consists of several base stations, which act as a gateway for data transmission to a remote observer.

**Figure 7.**
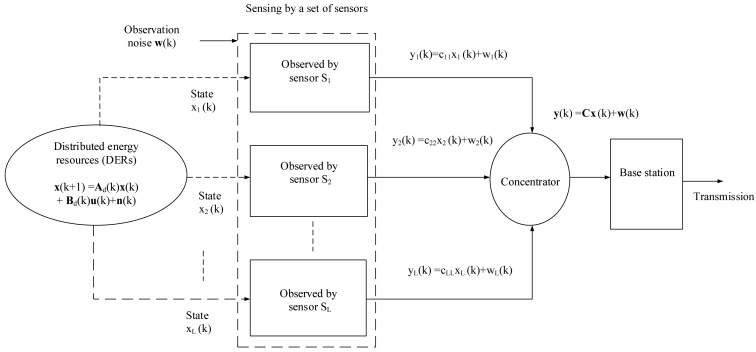
A linear decentralized WSN based DER monitoring system.

This paper considers a distributed structured wireless sensor network (WSN) with a concentrator where a set of sensors are deployed to get DER information. A linear decentralized WSN based DER monitoring system is demonstrated in Figure 7. All of the sensors communicate directly to a concentrator, which acts as a collector and a forwarder. It is assumed that there are no inter-sensor communication links, since the sensors are distributed [1,44].

**Figure 8.**
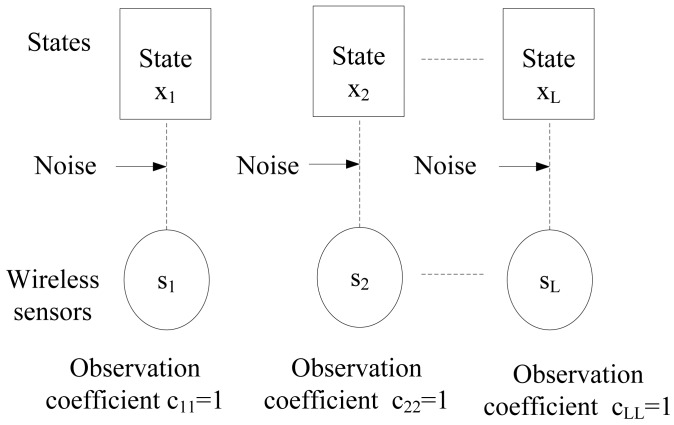
The bipartite graph for one sensor that can sense one system state directly.

The sensor *S_i_* is connected to the DER state variable *x_j_*, if *S_i_* observes the DER state variable *x_j_* directly [45]. For instance, the bipartite graph for the local observation matrix is shown in Figure 8.

Therefore, the observations from the multiple DER state information are obtained by a set of sensors as follows:

(7) y(k)=Cx(k)+w(k)

where **y**(*k*) is the observation information, **C** is the observation matrix, which maps the true state space to the observed space, and **w**(*t*) is the zero mean observation noise, whose variance is Σ*_w_*. The observation noise comes from sensing the states by using smart sensors, which are easy to deploy, low cost and flexible [46]. The proposed smart grid communication is illustrated in the next subsection.

### 3.4. Proposed Communication Systems for the Smart Grid

**Figure 9.**
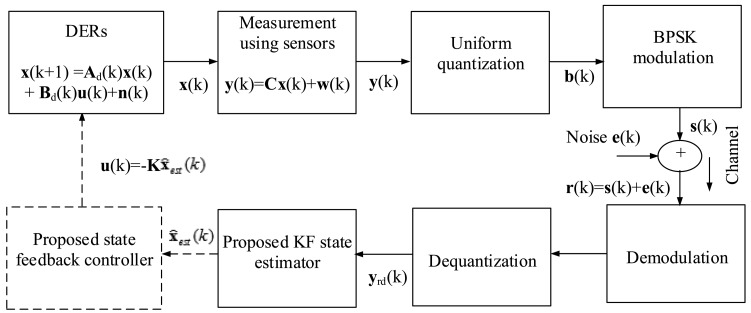
Model for communication systems and dynamic state estimation.

The observation information by the WSN is transmitted to the nearby base station. After that, the uniform quantizer of this base station maps each observation signal to a sequence of bits. To transmit the sensors bit sequence to the observer at the SCADA system, this paper uses binary phase shift keying (BPSK) as a modulation technique. The bit sequence **b**(*k*) is passed through a BPSK, and the modulated signal **s**(*k*) is obtained. The modulated signal goes through an additive white Gaussian noise (AWGN) channel. To illustrate, Figure 9 shows the communication procedure and dynamic SE.

The received signal at the supervisory control and data acquisition (SCADA) system is given by:

(8) r(k)=s(k)+e(k)

where **e**(*k*) is AWGN noise whose mean is zero and variance is Σ*_e_*. Then, the received signal is followed by demodulation, dequantization and, finally, fed to the proposed accuracy-dependent KF-based feedback controller for this DER dynamic system. This is the first contribution for sensing, estimating and controlling the DER states in such a way. Based on the proposed infrastructure, a state observer and DLQR control are proposed and implemented for the smart grid with communication systems. The proposed KF-based DER SE and feedback control method are described in the next section.

## 4. Proposed KF-Based DER SE and Feedback Control Method

This section tries to answer the following questions: (i) What is the optimal smart grid SE method for the multiple DERs? (ii) Based on the estimated states, what type of feedback control technique is applied for controlling the state of the multiple DERs? This paper attempts to answer these questions by presenting accuracy-dependent consensus steps in the standard KF SE method. The proposed approach is able to properly estimate the DER states through minimizing the estimation error based on the current estimation, learning rate parameter and trust covariance matrix. Finally, this paper proposes a DLQR to control the state of the microgrid incorporating multiple DERs. The following subsections describe the proposed accuracy-dependent KF and DLQR feedback control law for controlling the multiple DERs states in the smart grid.

### 4.1. Proposed KF-Based SE for Multiple DERs

The discrete time KF is a set of mathematical equations that provide an efficient recursive means to estimate the state of a process in a way that minimizes the mean square error (MSE). This algorithm works in two steps (prediction and correction step). In the prediction step, the KF estimates the current state variables along with their uncertainties [47]. In the correction step, the predicted estimation is further updated to get the desired estimate states. As can be seen in Figure 9, the demodulated output bit sequences are dequantized to obtain the sample states **y***_rd_*(*k*). The following intermediate steps are computed as follows:

(9) $${\hat{\text{x}}}^{-}(k)={\text{A}}_{d}\hat{\text{x}}(k-1)+{\text{B}}_{d}\text{û}(k-1)$$

where **x̂**^−^ (*k*) is the predicted state estimate and **x̂**(*k* − 1) is the initial given state vector. The predicted estimate covariance matrix is given by:

(10) $${\text{P}}^{-}(k)={\text{A}}_{d}\text{P}(k-1){\text{A}}_{d}^{T}+{\text{Σ}}_{nd}$$

where **P**^−^ (*k*) is the predicted estimate covariance matrix and **P**(*k* − 1) is the initial given covariance matrix. The updated state estimate (correction step) is given by:

(11) $$\hat{\text{x}}(k)={\hat{\text{x}}}^{-}(k)+\text{K}(k)[{\text{y}}_{rd}(k)-\text{C}{\hat{\text{x}}}^{-}(k)]$$

where the Kalman gain **K**(*k*) is given by:

(12) $$\text{K}(k)={\text{P}}^{-}(k){\text{C}}^{T}{\left[{\text{CP}}^{-}(k){\text{C}}^{T}+{\text{Σ}}_{w}\right]}^{-1}$$

and the updated estimate covariance matrix is given by:

(13) $$\text{P}(k)={\text{P}}^{-}(k)-\text{K}(k){\text{CP}}^{-}(k)$$

In order to improve the system performance, this paper proposes an accuracy-dependent consensus step together with KF steps [48]. It can regenerate the sample system state based on the above intermediate estimated state together with **A***_d_* and **B***_d_*. From this point of view, the proposed accuracy-dependent consensus steps are as follows:

(14) $${\hat{\text{x}}}_{\text{con}}(k)={\text{A}}_{d}\hat{\text{x}}(k)+{\text{B}}_{d}\text{u}(k)$$

(15) $${\hat{\text{x}}}_{\text{est}}(k)=\hat{\text{x}}(k)+\epsilon [{\hat{\text{x}}}_{\text{con}}(k)-\hat{\text{x}}(k)]$$

where *ϵ* is the learning rate, whose value ranges from zero to one and **x̂***_est_*(*k*) is the final estimated states of the real microgrid incorporating DERs. In order to improve the system performance based on the **x̂***_est_*(*k*), this paper proposes to update the above covariance matrix, which refers to the trust covariance matrix. Therefore, the proposed trust covariance matrix for KF is given by:

(16) $${\text{P}}_{kf}(k)=\text{P}(k)/tr[\text{P}(k)]$$

where *tr*(.) is the standard trace operator. Then, the above estimate covariance matrix **P**(*k*) is replaced by **P***_kf_*(*k*) in the next iterations. The following subsection presents the feedback control law to regulate the DER states.

### 4.2. Proposed Feedback Control Law

In order to regulate the DER state, define the following LQR feedback control law [41]:

(17) $$\text{u}(k)=-{\text{K}}_{fd}{\hat{\text{x}}}_{\text{est}}(k)$$

by minimizing the following cost function:

(18) $$J=\overset{N}{\underset{i=1}{\text{∑}}}\left[{{\hat{\text{x}}}^{\prime }}_{\text{est}}(i)\text{Q}{\hat{\text{x}}}_{\text{est}}(i)+{\text{u}}^{\prime }(i)\text{Ru}(i)\right]$$

where **Q** and **R** are positive-definite state weighting matrices (depending on DER system penalties for state deviations at different buses) and the control weighting matrix (depending on different DER operating costs). The physical meaning of the cost function is given below:

(1) The first term **x̂′***_est_***Qx̂***_est_* represents the weighted norm of the DER system state. The weight **Q** is used to control some states more tightly than other states.
(2) The second term **u′Ru** is the weighted norm of the control action, which represents the cost penalty on the control input. The weight **R** is used to maintain the amount of control action.
(3) The cost function is the weighted sum of the norms of the DER state variables and control action, so that we can balance between DER state deviations and control action.

Based on the above cost function, the LQR feedback control gain **K***_fd_*(*k*) is given by:

(19) $${\text{K}}_{fd}(k)={\left({\text{B}}_{d}{\text{FB}}_{d}^{T}+\text{R}\right)}^{-1}{\text{B}}_{d}^{T}{\text{FA}}_{d}$$

where **F** is the unique stabilizing solution of the discrete-time algebraic Riccati equation:

(20) $$\text{F}={\text{A}}_{d}^{T}{\text{FA}}_{d}+{\text{A}}_{d}^{T}{\text{FB}}_{d}{\left({\text{B}}_{d}{\text{FB}}_{d}^{T}+\text{R}\right)}^{-1}{\text{B}}_{d}^{T}{\text{FA}}_{d}+\text{Q}$$

As was mentioned, the future of smart grids is based on grid-integrated real-time communications between the grid elements of generation, transmission, distribution and loads [1]. In other words, one of the objectives of the smart grid is to increase the penetration of renewable energy sources in the generation cycle. The generation can be located in remote areas and operated in harsh environments, where continuous monitoring using low-cost sensors becomes necessary Therefore, it is intrinsically true that the smart monitoring system is expected to be adaptive, mobile in nature and flexible in topology, which can process different data, such as current, voltage, acoustic or radio frequency signals, and allow the multidirectional flow of information [1,2]. Therefore, WSNs have been considered as a promising technology that can enhance various aspects of today's electric power systems, making them a vital component of the smart grid. From the control perspective, the optimization process has two stages, so there is a two-way handshaking between the observer and sensors in smart grid control.

**Figure 10.**
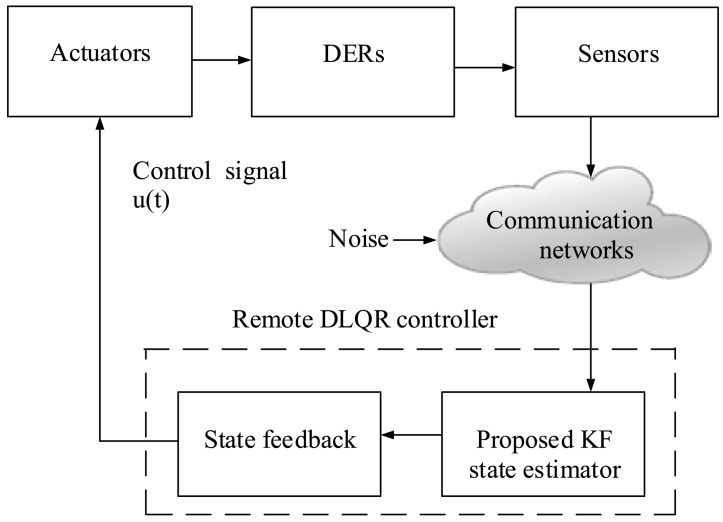
The role of the WSN in the optimization of the smart grid control.

As shown in Figure 10, a controller together with sensors and actuators is usually used to sense and control the DER states, compare it against the desired behaviour, compute control commands and perform actions on the system to achieve the desired change. Firstly, the DER data gathered by a set of sensors is transmitted directly to the corresponding base station via single-hop communications. Efficient aggregation of data collected by sensors is crucial for successful WSN-based smart grid applications. Secondly, the smart control centre processes all incoming data by executing pre-designed accuracy-dependent KF and DLQR control. Smart actuators in WSNs perform appropriate actions to maintain network stability and the operation of the DERs. Thus, WSNs offer an ideal technology for the monitoring and control of the generation facilities in smart grids. The simulation results of the proposed KF-based DER SE and feedback control method are demonstrated in the next section.

## 5. Simulation Results

In this section, we examine the proposed accuracy-dependent KF and DLQR controller numerically based on the DER models. We consider a microgrid model incorporating three DERs, which are sensed by a set of sensors. The system state is an 11-dimensional vector. The DERs are connected to the IEEE three-bus distributed system operated in island mode. The simulation parameters of the smart grid are summarized in Table 1.

### 5.1. DERs State Estimation Results

**Figure 11.**
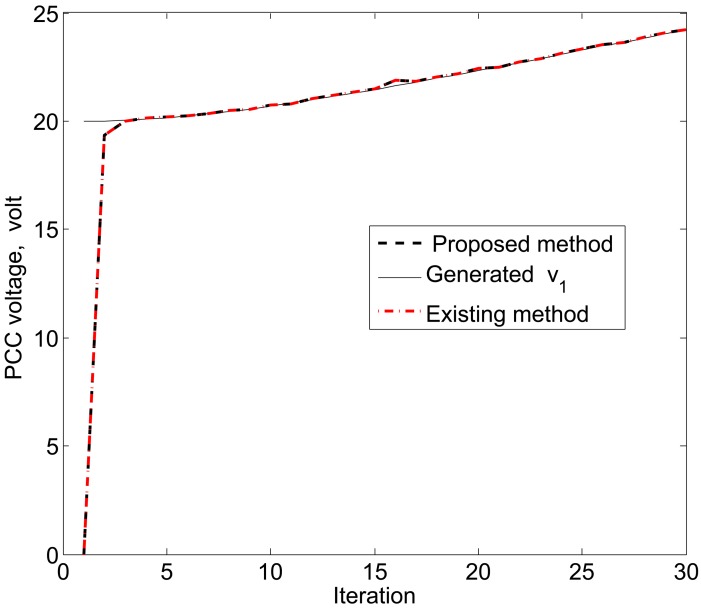
Point common coupling (PCC) voltage estimation for *υ*_1_ using the proposed KF SE.

**Figure 12.**
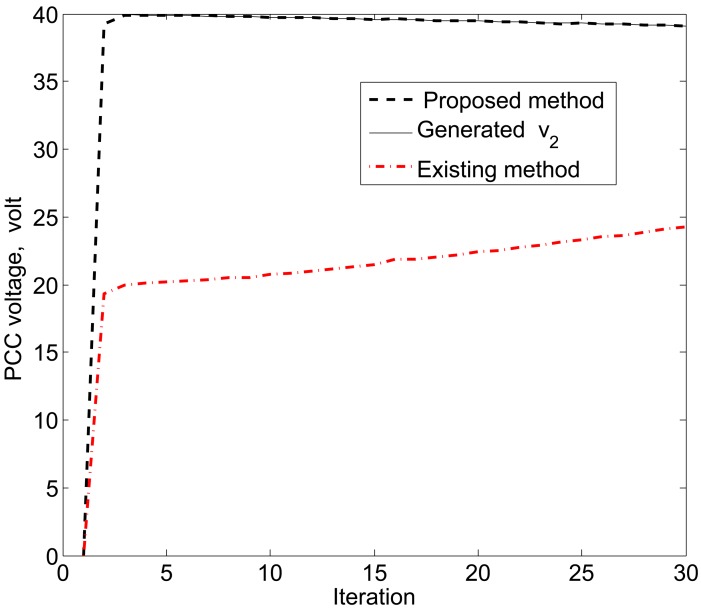
PCC voltage estimation for *υ*_2_ using the proposed KF SE.

**Figure 13.**
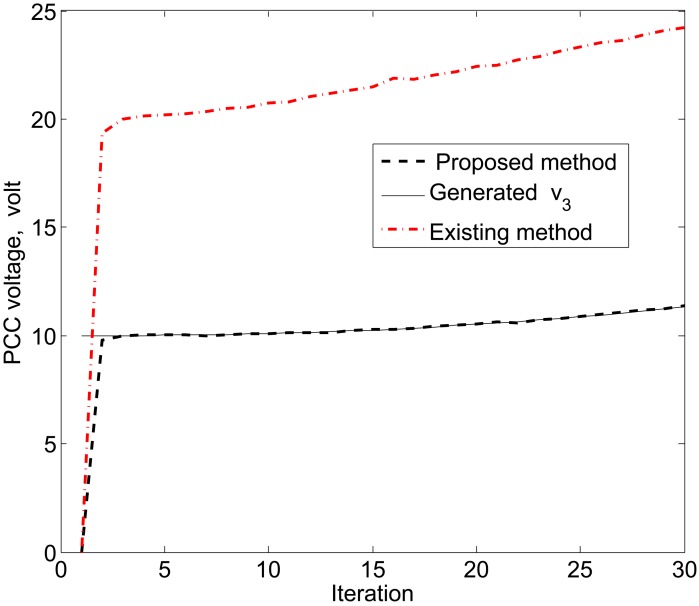
PCC voltage estimation for *υ*_3_ using the proposed KF SE.

The simulation results in Figures 11, 12 and 13 reveal that the proposed KF is able to estimate the system state properly. This accurate estimation is obtained using the proposed accuracy-dependent consensus steps in the standard KF. Note that the small fluctuations come from the process, observation and channel noises.

**Figure 14.**
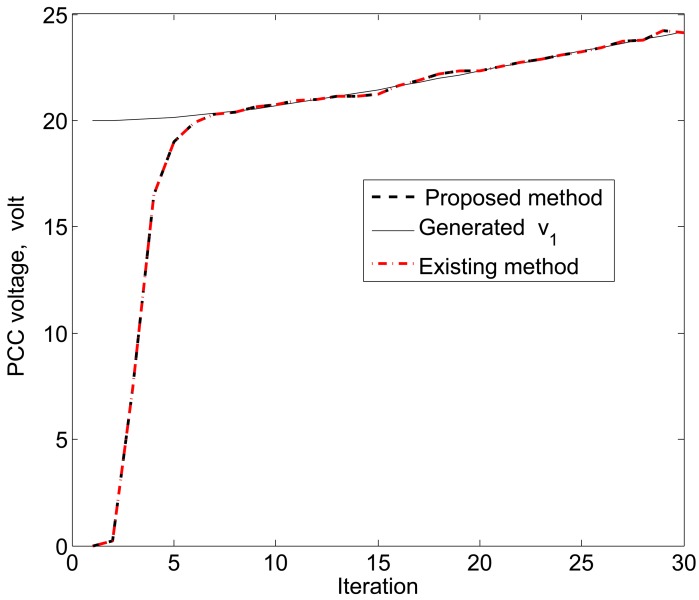
PCC voltage estimation for *υ*_1_ using the proposed KF SE with two faulty sensors.

**Figure 15.**
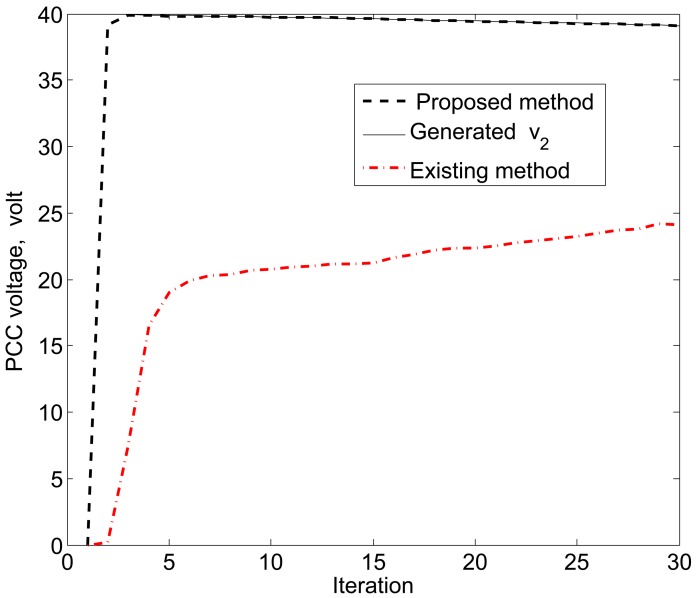
PCC voltage estimation for *υ*_2_ using the proposed KF SE with two faulty sensors.

**Figure 16.**
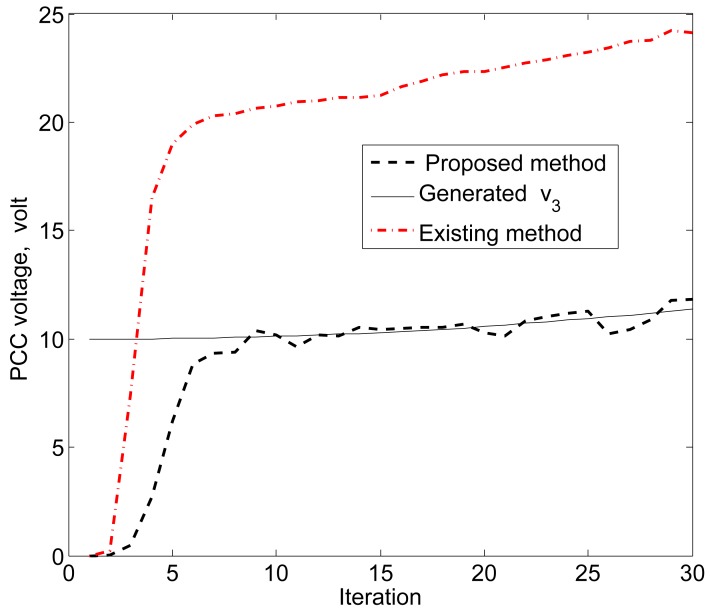
PCC voltage estimation for *υ*_3_ using the proposed KF SE with two faulty sensors.

For simplicity, we consider three DERs, so there are a total of 11 DER states. Furthermore, it is assumed that the smart sensors can sense the states directly to form an observer model. Therefore, the observations from the multiple DER states’ information are obtained by 11 smart sensors. However, in practical scenarios, there is a possibility that the sensor battery becomes low, so it cannot sense the system state properly. In this case, it is assumed that two sensors in the position of PCC voltages *υ*_1_ and *υ*_3_ are out of order among the 11 sensors. From the simulation results, as shown in Figures 14, 15 and 16, it can be seen that the proposed KF is able to estimate system states properly. Obviously, it needs a few more iterations to track the DER states compared with the 11-sensor case. Due to these sensing problems, the PCC voltages *υ*_1_ and *υ*_3_ cannot be sensed by the corresponding sensors. Interestingly, the proposed accuracy-dependent KF is able to track these states with steady-state errors. Nevertheless, the *υ*_2_ is directly sensed by the sensor, so it has a similar estimation result compared with the 11-sensor case. The obtained results indicate that it is better to use the same number of sensors as the states to properly estimate the DER states in smart grids.

### 5.2. Controlling the DER States

**Figure 17.**
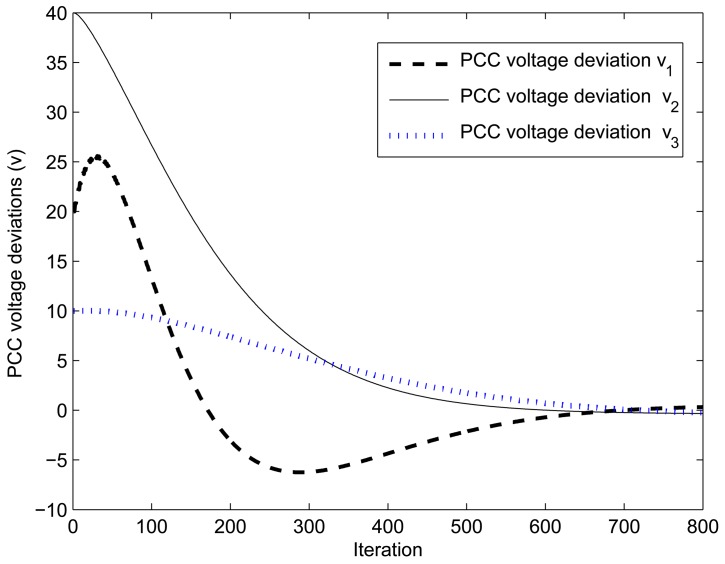
PCC voltage control using the proposed control method.

From the simulation results, as shown in Figure 11, it is noticed that the PCC voltage deviations increase dramatically, which is very dangerous in terms of network stability and the operation of DERs. Therefore, it is necessary to apply a proper control method, so that the PCC voltage deviations are driven to zero. This paper proposes a DLQR to control the DER state. As the DER system is fully observable, the proposed KF is able to estimate the system states properly. Then, the estimated system states are fed back to the DER for controlling the PCC voltage deviations. It can be seen in Figure 17 that the proposed DLQR controller is able to keep PCC voltage deviations to zero in approximately seven seconds (step size × iteration). With the reliable SE result, one can control the PCC voltage level, monitor the system performance, find out the optimal meter placement and analyze the malfunction of both the monitoring system and the grid.

## 6. Conclusions and Future Work

In order to design a green smart control center, this paper proposes a novel approach to estimate and control the real microgrid states. To achieve these goals, this paper proposes an accuracy-dependent consensus scheme in the standard KF steps and a DLQR method to control the DER state. Therefore, integrating these two approaches with application to the smart grid forms a novel contribution in the green energy and control research communities. The simulation results demonstrate that the proposed KF-based microgrid SE and control algorithm provides accurate SE and control compared with the existing KF. Although our results of the accuracy-dependent KF and DLQR scheme are promising for smart grid applications, the work presented here has some limitations. Therefore, further investigations include the following aspects:

- Consider more generic communication infrastructures, such as multihop and multicast ones, as well as the inter-DER communication links. Moreover, due to the interference among the sensors, scheduling is necessary for the transmission of the sensing information [3]. In this case, each sensor multicasts its observation to the multiple DERs, and these DERs can communicate with each other to coordinate the estimation and control. Therefore, a distributed scheduling algorithm is necessary within the framework of DER hybrid dynamical systems.
- The considered central estimator can be used if there are no channel bandwidth limitations, there is enough processing power in the measurement devices and there are enough computer resources to process the huge amount of measurements. In other words, a real-time central estimator in large-scale power systems with thousand of sensors is almost impossible due to the processing power limitations, networks congestion and security issues [34]. From this point of view, it is more desirable to develop distributed approaches to obtain the system-wide state estimates through limited information exchange among the multiple system operators. Therefore, an alternative approach is required to monitor such foreseeable resources.
- To meet the stringent latency requirements, the communication and power infrastructures have to collaborate strongly in smart grids. Therefore, this will require a combined process for the design and simulation of these networks that incorporate the actual WSNs and investigating the effects on the performance of these networks in terms of delay, throughput, power consumption and computational capabilities.
- Implement the communication networks considering more realistic scenarios, such as radio frequency disturbances, noise spikes and fading, that can be easily experienced by the wireless nodes.
- Extend the proposed methodology to incorporate the recursive systematic convolutional channel coding scheme for protecting and redundancy in the DER messages under the Rayleigh fading channel condition.

## Figures and Tables

**Table 1.** The system parameters (Ohm and Amp) for simulations.

**Parameters**	**Values**	**Parameters**	**Values**
R*_l_*_1_	76	R*_l_*_2_	76
R*_l_*_3_	76	R_1_	76
R_2_	76	R_3_	76
i*_l_*_1_	76	i*_l_*_2_	76
i*_l_*_3_	76	R*_t_*_1_	1
R*_t_*_2_	5	R*_t_*_3_	10
i*_t_*_1_	0.1	i*_t_*_2_	10
i*_t_*_3_	100	R*_d_*_1_	1.5
R*_d_*_2_	6	R*_d_*_3_	10
i*_d_*_1_	300	i*_d_*_2_	900
i*_d_*_3_	1500	C_1_	0.9
C_2_	0.9 F	c_3_	0.9
Δ*t*	0.01	Learning rate	0.2
Slots	1000	Quantization bits	16
Σ*_n_d__*	0.00001 × **I**	Σ_*w*_	0.001 × **I**
